# Immunohistochemistry Staining-Proven Cytomegalovirus Colitis in Living Donor Liver Transplantation

**DOI:** 10.3390/v15010115

**Published:** 2022-12-30

**Authors:** Shu-Hsien Lin, Kun-Ta Wu, Chih-Chi Wang, Ting-Ting Liu, Hock-Liew Eng, King-Wah Chiu

**Affiliations:** 1 Division of Hepato-Gastroenterology, Department of Internal Medicine, Kaohsiung Chang Gung Memorial Hospital, Taiwan 123 Ta-Pei Road, Niao-Sung District, Kaohsiung 833, Taiwan; 2 Liver Transplantation Program, Kaohsiung Chang Gung Memorial Hospital, Kaohsiung 833, Taiwan; 3 Division of General Surgery, Department of Surgery, E-Da Hospital, Kaohsiung 833, Taiwan; 4 Division of General Surgery, Department of Surgery, Kaohsiung Chang Gung Memorial Hospital, Kaohsiung 833, Taiwan; 5 College of Medicine, Chang Gung University, Taoyuan 333, Taiwan; 6 Department of Pathology, Kaohsiung Chang Gung Memorial Hospital, Kaohsiung 833, Taiwan

**Keywords:** cytomegalovirus colitis, immunoglobulin, inclusion body, living donor liver transplantation

## Abstract

**Background and Aims:** Cytomegalovirus (CMV) infection is a common occurrence in liver transplantation (LT) even in an era of preventive strategies. However, the diagnosis of CMV colitis remains challenging. This study aimed to focus on the clinical significance of endoscopic biopsy-proven CMV colitis in patients following living donor liver transplantation (LDLT). **Methods:** From January 2007 to December 2021, a total of 55 CMV colitis cases were retrospectively enrolled and divided into a non-LDLT group in 53 and an LDLT group in 2 cases. Clinical demographics, diagnostic measurement, histopathology, and anti-viral therapy were investigated. **Results:** There were 1630 cases undergoing LDLT in the period 2007–2021, with only 2 recipients being confirmed to have CMV colitis in 2021 (2/114, 1-year incidence: 1.75%). Comparisons between the 53 non-LDLT cases and 2 LDLT cases are as follows: Serum anti-CMV immunoglobulin M (IgM) was shown to be positive (n = 3, 5.5% vs. n = 0, *p* = 1.0) and negative (n = 20, 37.7% vs. n = 2, 100%, *p* = 0.16); anti-CMV immunoglobulin G (IgG) was positive (n = 19, 35.8% vs. n = 2, 100%, *p* = 0.14) and none were negative; CMV DNAemia was shown to be detectable (n = 14, 26.4% vs. n = 1, 50%, *p* = 0.47) and undetectable (n = 14, 26.4% vs. n = 1, 50%, *p* = 0.47). Among the two recipients with CMV colitis, one had CMV DNAemia and the other had no CMV DNAemia upon the development of symptoms; negative anti-CMV-IgM and positive anti-CMV-IgG were observed both pre-transplant and post-transplant; finally, CMV colitis was documented based on the presence of inclusion bodies and positive immunohistochemistry (IHC) staining in histology. **Conclusion:** Patients with immunocompromised status, in particular organ transplantation, may have positive serum anti-CMV IgM/IgG antibodies both before and after transplantation. This study emphasized the fact that endoscopic biopsy with IHC staining may be a more powerful tool for making an accurate diagnosis of CMV colitis in the setting of living donor liver transplantation.

## 1. Introduction

Liver transplantation (LT) is a useful strategy with which to extend life expectancy and improve the quality of life for patients with end-stage liver disease [1]. However, these recipients have increased risk of opportunistic infections due to the use of intense immunosuppressive therapy. Among the many pathogens of viral infection that commonly infect LT recipients, cytomegalovirus (CMV) remains the most significant cause of substantial morbidity and mortality [2,3,4,5]. In the last 2 years, German liver transplant centers have conducted a series of discussions, summarizing the optimal preventive and therapeutic measures for CMV infections after liver transplantation [6]. Even with the pre-transplant screening of CMV serology from donors and transplant candidates as well as the widespread implementation of CMV prevention strategies, CMV disease continues to occur following transplant [7]. Particularly, CMV colitis, the most frequently affected site of tissue invasive-GI CMV disease [8,9,10], is rarely discussed in living donor liver transplant (LDLT) recipients.

According to the definition of CMV disease in transplant patients from the infectious diseases society of America (IDSA) guidelines, a “proven” CMV gastrointestinal (GI) disease requires symptoms of GI tract plus macroscopic mucosal lesions plus CMV documented in tissue by histopathology [11]. Although previous research has reported that a lower pre-transplant anti-CMV immunoglobulin G (IgG) titer is significantly associated with CMV infection after LT and that pre-transplant anti-CMV IgG levels could prevent post-transplant severe CMV infections in LT recipients [12], the role of the anti-CMV immunoglobulin level in the diagnosis of CMV disease with GI tract involvement has seldom been discussed. Determining true GI tract CMV disease is still challenging and great efforts are required to avoid underestimation.

CMV is the most common infectious complication following liver transplantation. Early detection and prompt treatment for CMV disease are warranted in order to improve graft survival. In this retrospective study, we compared the difference between patients with CMV DNAemia vs. non-DNAemia and LDLT vs. non-LDLT in terms of clinical manifestations, serology test, as well as anti-viral treatment; we also demonstrated two cases of CMV colitis following LDLT from real-world experience in our liver transplantation program. Herein, we aimed to emphasize the endoscopic biopsy with typical histological examination as one of the significantly accurate detection methods for the diagnosis of CMV colitis, the most frequently affected site of tissue invasive-GI CMV disease in LDLT recipients.

## 2. Methods

### 2.1. Study Population and Design

By retrospectively searching the pathology database of Kaohsiung Chang Gung Memorial Hospital, a living donor liver transplant center in Taiwan, from January 2007 to December 2021, we found a total of 55 documented CMV colitis cases.

Overall, there were 1630 cases of patients undergoing LDLT in this 15-year retrospective study period. In the year of 2021, there were 114 LDLT recipients, with only 2 cases confirmed to have CMV colitis via histologic examination (2/114, 1-year incidence: 1.75%). Additionally, no CMV colitis recipients with a history of other organ transplantation such as kidney or lung transplantation were documented.

The following clinical parameters were recorded from the medical charts of all the patients with biopsy-proven CMV colitis: age, gender, underlying disease, clinical manifestations, location of lesion, laboratory tests (serum anti-CMV IgM, anti-CMV IgG, and CMV PCR DNA amplification assays) in the interval of 2 weeks before or after the date of diagnosis, histopathologic findings, treatment, and complications.

In this study, all of the patients with definite CMV colitis were further divided into subgroups: CMV DNAemia group vs. CMV non-DNAemia group and LDLT group vs. non-LDLT group. The differences in regard to clinical characteristics, serologic results, histopathologic features, and treatment were investigated and compared in these subgroups. Finally, we also marked the two patients with a history of LDLT in order to emphasize the importance of colon biopsy to all clinically suspicious CMV colitis cases after LDLT.

### 2.2. Data Collection and Diagnostic Assessment

All recipients received the same immunosuppression protocol of our liver transplantation program after LDLT. Serum biochemistry tests were routinely monitored for evaluating graft function. The following data of the 2 post-LDLT recipients with definite CMV colitis were collected: demographics (gender and age), primary liver disease before LDLT, clinical manifestations, laboratory tests, endoscopic abnormalities, histopathologic features, treatment, and outcomes. Laboratory tests included complete blood cell count and C-reactive protein (CRP) from recipients, serum anti-CMV IgM, anti-CMV IgG, as well as CMV DNA PCR from donors and recipients.

Anti-CMV IgM and IgG antibody titers were measured with chemiluminescent microparticle immunoassay (CMIA). Anti-CMV IgM and IgG reports were interpreted as follows: negative (<0.85 Index) and negative (<6.0 arbitrary units (AU)/mL), respectively. Serum CMV DNA quantitative amplification test was measured via quantitative real-time polymerase chain reaction (real-time qPCR). The DNA load was reported in IU/mL. The lower limit of quantification was <34.5 IU/mL.

Tissue specimens were obtained from the base of abnormal mucosal lesion by using colonoscopy biopsy forceps in multiple sessions. Histopathologic features of the biopsy specimens from the colonic ulcers or erosions specified focused on the detection of virus inclusion bodies by routine hematoxylin-eosin (H&E) staining and immunohistochemistry (IHC) staining using monoclonal antibodies directed against the CMV pp65 antigen (Novocastra™ lyophilized mouse monoclonal antibody; Leica Microsystems, Wetzlar, Germany). The medication history and antiviral therapy (ganciclovir or valganciclovir) used were also recorded. Post-transplantation outcome was evaluated.

### 2.3. Ethical Statement

All procedures involving human participants were performed in accordance with the ethical standards of the institutional committee and with the 1964 Helsinki Declaration and its later amendments or comparable ethical standards. The study protocol was approved and authorized by the Medical Ethics Committee of Chang Gung Memorial Hospital, Kaohsiung (ethical approval number: 202002159B0C502). No allograft donor or recipient was from a vulnerable population.

### 2.4. Statistical Analysis

SPSS (version 22.0; SPSS Inc., Chicago, IL, USA) was used for data analysis. Descriptive values are expressed as mean ± standard deviation (SD) and percentages. Categorical variables were compared using the chi-square or Fisher’s exact test, and continuous variables were compared using Student’s *t*-test. All tests were two-tailed, and a *p* value of <0.05 was considered significant.

## 3. Results

### 3.1. Demographics and Clinical Characteristics of all Patients with Biopsy-Proven CMV Colitis

**Figure 1:**
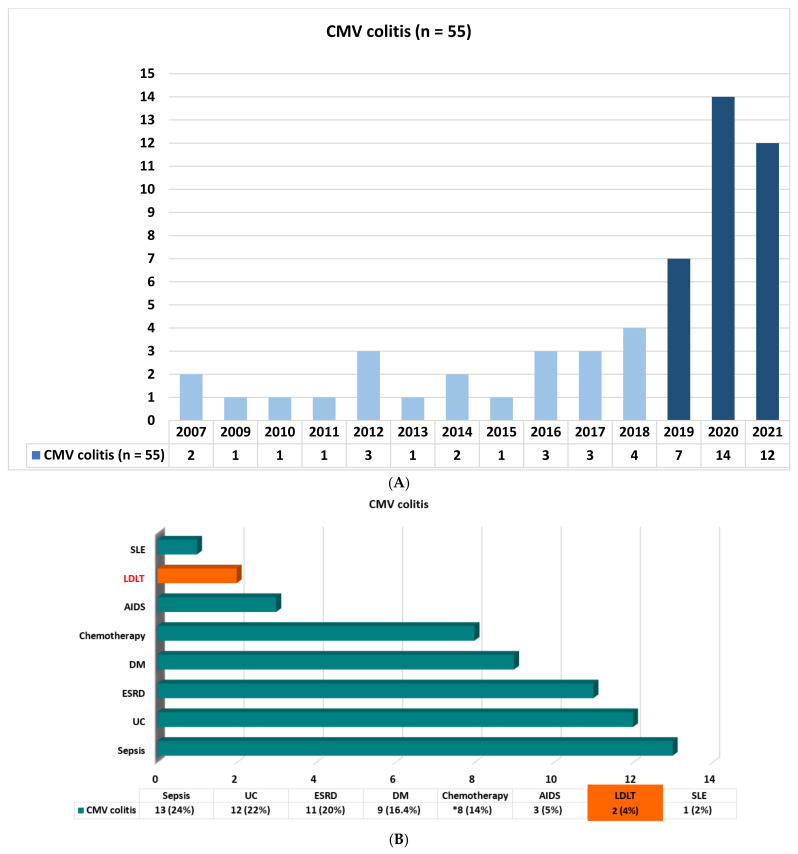
(**A**) Biopsy-proven cytomegalovirus colitis from 2007 to 2021. (**B**) Demographic characteristics of all patients with biopsy-proven cytomegalovirus colitis. UC: ulcerative colitis; ESRD: end stage renal disease; DM: diabetes mellitus; AIDS: acquired immunodeficiency syndrome; LDLT: living donor liver transplantation; SLE: systemic lupus erythematosus; *: chemotherapy for colon cancer (n = 2, 3.6%), cancer of non-gastrointestinal tract (n = 6, 10.9%).

From January 2007 to December 2021 in Kaohsiung Chang Gung Memorial Hospital, a total of 1630 cases underwent LDLT in the period 2007–2021, with only two recipients confirmed to have CMV colitis via histologic examination in 2021 (2/114, 1-year incidence: 1.75%). In this 15-year study period, a total of 55 cases with endoscopic biopsy-proven cytomegalovirus colitis were enrolled for analysis and comparison: 33 cases (60%) were diagnosed in the period 2019–2021 (Figure 1A). The underlying associated diseases of all patients in this cohort were as follows: sepsis (n = 13, 24%), ulcerative colitis (n = 12, 22%), end-stage renal disease (n = 11, 20%), diabetes mellitus (n = 9, 16.4%), chemotherapy for colon cancer/cancer of non-gastrointestinal tract (n = 2, 3.6%/n = 6, 10.9%), acquired immunodeficiency syndrome (n = 3, 5%), living donor liver transplantation (n = 2, 4%), and systemic lupus erythematosus (n = 1, 2%) (Figure 1B).

The clinical characteristics, laboratory test, pathology findings, and treatment of all patients with biopsy-proven cytomegalovirus colitis are shown in Table 1. Among the 55 eligible patients, the mean age was 68 years. Male gender and female gender were 28 cases (50.9%) and 27 cases (49.1%), respectively. Bloody stool (n = 35, 63.7%) was the most common clinical symptom, followed by diarrhea (n = 13, 23.6%). Colonoscopy examination illustrated the rectum (n = 24, 43.6%) as the most frequently involved location of abnormal lesions. In the interval of 2 weeks before or after the date of definite diagnosis, serology testing of anti-CMV IgM was shown to be positive in only 3 cases (5.5%) and negative in 22 cases (40%); anti-CMV IgG was shown to be positive in 21 cases (38.2%) and negative in 0 cases (0%). Among the 55 patients, serum CMV DNA was detectable in 15 patients (27.3%), and undetectable in 15 patients (27.3%). Histopathological examination revealed the positive CMV IHC staining in all of the patients (n = 55, 100%), and the presence of inclusion bodies in 32 cases (58.2%). A total of 17 cases (30.9%) were receiving single anti-viral agent with intravenous ganciclovir (5 mg/kg/dose Q12H) and 12 cases (21.8%) were receiving oral valganciclovir (900 mg Q12H) only; a combination regimen with initial intravenous ganciclovir (5 mg/kg/dose Q12H), followed by oral valganciclovir (900 mg Q12H), was administered in 10 patients (18.2%). Complications associated with CMV colitis (bowel perforation and bowel ischemia) developed in two cases (3.6%).

### 3.2. Comparison between CMV DNAemia and Non-CMV DNAemia

The clinical characteristics, underlying associated disease, serology test, pathology findings, and treatment used were compared between the two subgroups (CMV DNAemia vs. non-CMV DNAemia), as shown in Table 2. There was no statistically significant difference between the two subgroups.

### 3.3. Comparison of CMV Colitis between Patients with and without Living Donor Liver Transplantation

A comparison between the two subgroups of CMV colitis patients with (n = 53) and without (n = 2) a history of undergoing LDLT, in terms of clinical characteristics, serology test, pathology findings, and anti-viral therapy was made. There was no statistically significant difference between the two subgroups (Table 3).

### 3.4. CMV Colitis in Living Donor Liver Transplantation

**Figure 2:**
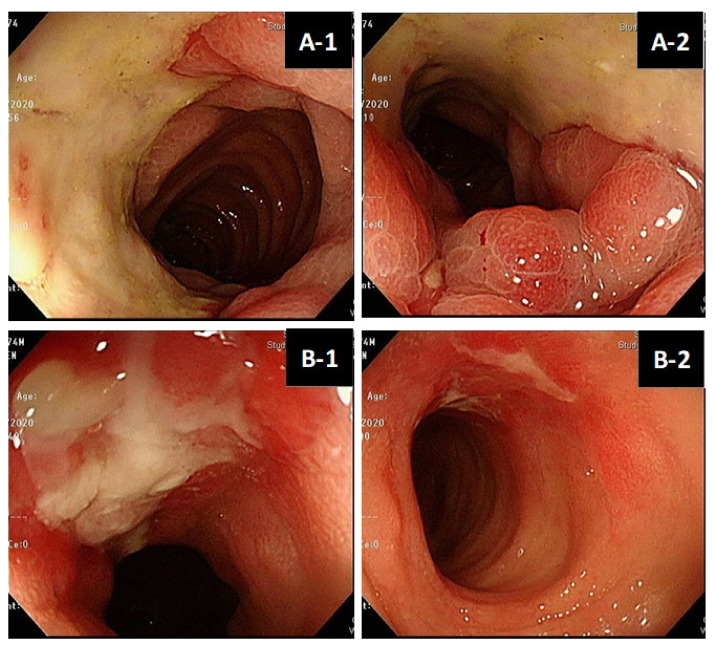

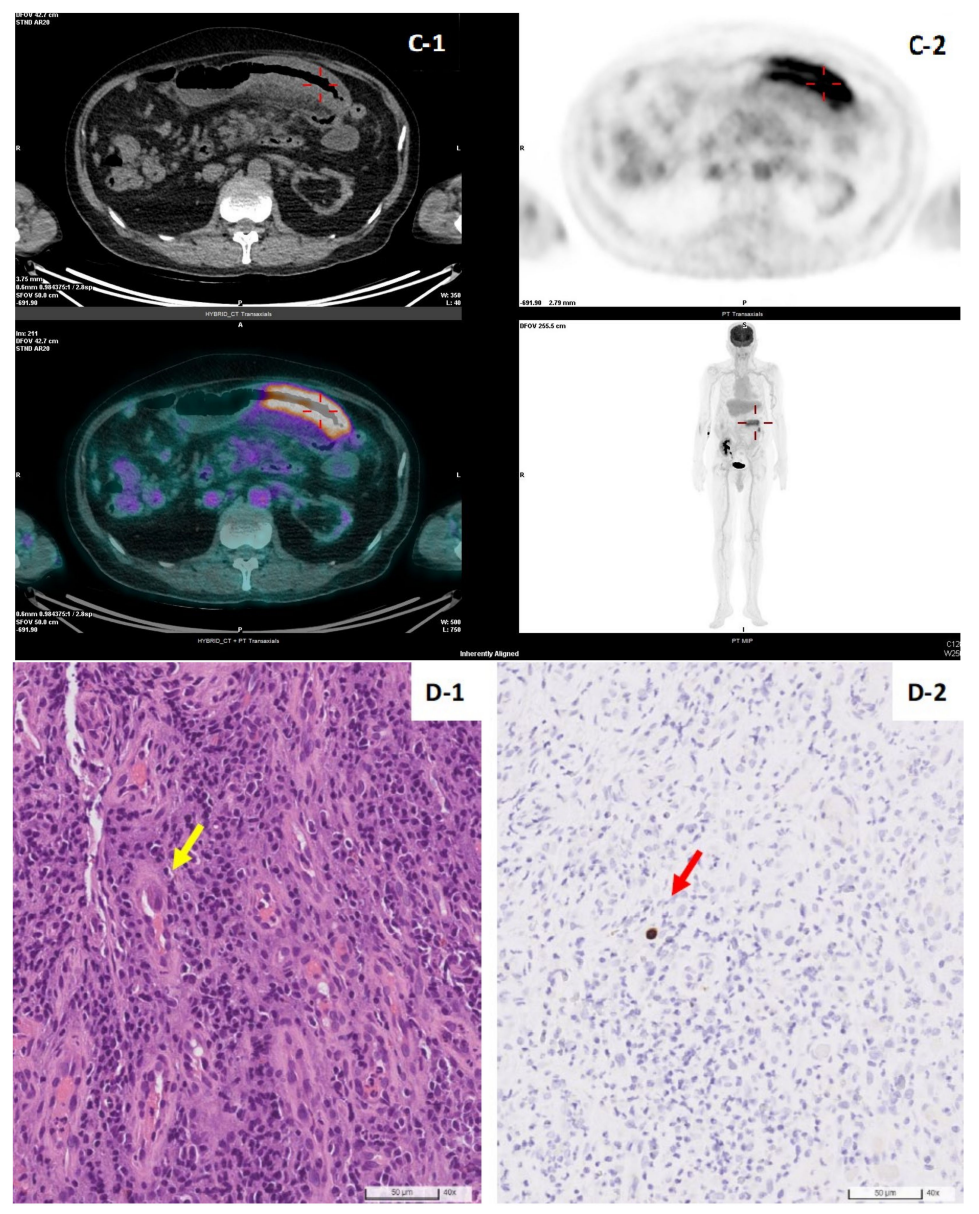
(**A**,**B**). Colonoscopy features of living donor liver transplant patient No.1. Well-demarcated longitudinal ulceration (around 3 cm in diameter) in descending colon with colonic mucosa edematous change and bowel wall thickening, causing intra-luminal narrowing (**A-1**,**A-2**). Remission of ulceration 2 weeks later after antiviral therapy follow-up (**B-1**,**B-2**). (**C**). Positron Emission Tomography/Computed Tomography (PET/CT) of patient No.1. PET/CT illustrated segmental colon wall thickening at distal T-colon and proximal D-colon by CT scan (**C-1**, arrow) and increased FDG uptake in the colon wall, SUV max:7.6 by PET (**C-2**, arrow), respectively. (**D**). Histopathology of CMV colitis in patient No.1. Histological hematoxylin and eosin staining (×40 objective) detection of CMV inclusion bodies (owl’s eye) (yellow-arrow), biopsy specimen of an ulcer at descending colon (**D-1**). Positive CMV immunohistochemistry (IHC) staining (×40 objective) (red-arrow) (**D-2**).

The patient profiles of CMV colitis after living donor liver transplantation are presented in Table 3. Recipient 1 was a 67-year-old male patient with a history of living donor liver transplantation for the reason of decompensated liver cirrhosis about 10 years ago. During the 10-year follow-up period after LDLT, he experienced the complication of moderate acute cellular rejection in the second year after LDLT. He used immunosuppressants with mycophenolate mofetil at 250mg Q12H PO and tacrolimus at 1mg QD PO at the time of diagnosis of CMV colitis. His clinical manifestations were abdominal pain and loose diarrhea for several weeks. Serum anti-CMV IgM before and after LDLT were both negative; serum anti-CMV IgG before and after LDLT were both positive. Serum CMV DNA PCR was undetectable. Colonoscopy demonstrated well-demarcated longitudinal ulceration (around 3 cm in diameter) in the transverse colon and descending colon with colonic mucosa edematous change and bowel wall thickening, causing intra-luminal narrowing (Figure 2A-1,A-2). Positron Emission Tomography/Computed Tomography (PET/CT) illustrated segmental colon wall thickening at distal transverse colon and proximal descending colon (Figure 2C-1, arrow) via CT scan and increased FDG uptake in the colon wall, with SUV max: 7.6 (Figure 2C-2, arrow) via PET, respectively. The histopathological examination of the biopsy specimen of an ulcer at descending colon disclosed CMV inclusion bodies (owl’s eye) (Figure 2D-1). Positive CMV immunohistochemistry staining (×40 objective) was detected (Figure 2D-2). Based on the typical presentation of CMV colitis in pathohistological examination obtained from colonic biopsy specimen, the diagnosis of CMV colitis was definitely confirmed. Initial intravenous ganciclovir (5 mg/kg/dose Q12H) was administered for 2 weeks, followed by oral valganciclovir (900 mg Q12H) for 3 months. Repeated colonoscopy on the second week after anti-viral therapy showed significant remission of previous ulcerations (Figure 2B-1,B-2).

In contrast to recipient 1, recipient No. 2 was diagnosed to have CMV colitis about 2 months after LDLT. She used immunosuppressants with mycophenolate mofetil 500 mg Q12H PO, Prednisolone 5 mg TID PO, and Tacrolimus 2mg QD PO at the time of diagnosis of CMV colitis. During the 2-month follow-up period after LDLT, she experienced a complication of the biliary tract stricture requiring endoscopic retrograde cholangiopancreatography with biliary stent. She had serum CMV DNAemia (123 IU/mL) at the time of diagnosis. Furthermore, this patient was also confirmed to have CMV colitis via colonic biopsy with the presence of inclusion bodies and positive immunohistochemistry staining in histology. Anti-viral therapy was prescribed with 172 days of treatment course.

## 4. Discussion

CMV colitis in patients with both immunocompromised status as well as organ transplantation is a critical problem which needs to be overcome. This 15-year retrospective study emphasizes the clinical significance of endoscopic biopsy for suspicious lesions for the diagnosis of CMV colitis in our hospital. This research was conducted in Taiwan’s tertiary medical center specializing in LDLT. However, only two cases with a history of LDLT were identified for further investigation. No CMV colitis recipients with a history of other organ transplantation such as kidney or lung transplantation were documented. A possible explanation for the rarity of CMV colitis in organ transplantation patients may be the underestimation of the true incidence due to the suboptimal diagnostic methods used. To the best of our knowledge, this is the first study to address the clinical significance of biopsy-proven CMV colitis recipients with and without DNAemia in patients undergoing LDLT.

In the literature, research on six cases of gastric biopsy-proven CMV gastritis conducted by Dan Chen et al. pointed out that endoscopic biopsy is the major diagnostic method for CMV gastritis [13]. Recently, Pai-Jui Yeh et al. reported on the clinical manifestations, risk factors, and prognostic factors of CMV enteritis [14], but they did not focus on patients with a history of LDLT. In our study, we identified a total of 55 patients with colon biopsy-proven CMV colitis in our hospital in the period 2007–2021. Sixty percent of the cases were diagnosed in the last 3 years, especially those with underlying ulcerative colitis disease (n = 12, 22%) (Figure 1A,B). In fact, six cases had moderate to severe UC disease activity. According to the previous review of the literature, CMV infection is found in 10–38% of patients with active UC. Patients with medically refractory UC could be prone to CMV infection because of their use of immunosuppressive drugs, especially corticosteroid, in addition to sustained inflammation in the colonic mucosa triggering CMV reactivation [15,16,17]. Only two cases with a history of LDLT were identified (Figure 1B); the reason for the small number of cases of CMV colitis in LDLT might be explained by both universal prophylaxis and the strategy of preemptive therapy for treating CMV infection as well as clinical underdiagnosis.

As shown in Table 1 and Table 2, serum anti-CMV IgM and IgG as well as serum CMV DNA qPCR assay were not diagnostically helpful; in contrast, tissue IHC staining from colon biopsies was all positive. These findings were compatible with previous research: blood serologic testing for CMV has no diagnostic value for CMV colitis since the seroprevalence of CMV within the adult population is high (at least 40% seropositive) [18,19]. Additionally, the neutrophil-to-lymphocyte (N/L) ratio, a novel marker of systemic inflammation, was considered a useful predictor for viral infections such as CMV, influenza, and COVID-19 [20,21,22,23]. In our study, a higher N/L ratio was found in the group of CMV DNAemia, although there was no statistical significance.

Despite the use of a small sample size of CMV colitis patients in LDLT, we demonstrated no statistically significant difference between the subgroups of Non-LDLT and LDLT in terms of their clinical characteristics and serum laboratory tests (Table 3). However, in order to put emphasis on the significance of an accurate diagnostic approach for CMV colitis after LDLT, we further explored the detailed profiles of the two recipients (Table 4). In our liver transplantation program, we followed the recommendations made by Western guidelines regarding the administration of post-transplant immunosuppressants and a CMV prevention strategy (prophylaxis or preemptive treatment) depending on the sero-status of the donor/recipient [1,3,6,7]. It was reported that a seropositive donor and seronegative recipient (D+/R−) match conferred the highest risk for CMV infection, with rates of 44–65% without prophylaxis, whereas D+/R+, D−/R+, and D−/R- status conferred rates of 18–20%, 7.9%, and 1–2%, respectively [16,24]. CMV D+/R− serogroup status remains independently associated with increased graft loss and mortality in LT recipients [25].

In our two cases, recipient No. 1 had sero-status (D+/R+) and undetectable CMV DNA at the time of CMV colitis diagnosed; by contrast, recipient No. 2 had sero-status (D−/R+) and detectable CMV DNA; both of them were confirmed to have CMV colitis via viral inclusion bodies in HE staining and positive IHC staining (Figure 2D) from biopsy to the colonic ulcer base (Figure 2A). These findings emphasize the fact that invasive endoscopic procedures for the pathological confirmation of CMV colitis cannot be replaced by blood serologic testing only. Negative serum CMV qPCR results do not exclude the possibility of tissue-invasive GI CMV disease [26,27].

Based on the results from our study, the diagnostic potential of anti-CMV IgM, IgG, and CMV DNA was limited for CMV colitis. Negative anti-CMV immunoglobulin or negative CMV DNA do not exclude the possibility of CMV colitis, a tissue-invasive CMV disease. Due to the incomplete data available in this retrospective study, we had difficulty in further analyzing the association between anti CMV immunoglobulin and CMV DNA with the diagnosis of CMV colitis in this small sample size. However, according to a previous study conducted by Jang EY et al., the sensitivity of the CMV antigenemia test for the diagnosis of CMV gastrointestinal disease was 54%, with a 95% confidence interval (41–68%) [28]. However, studies from Similan Kirisri et al. and Jackrapong Bruminhent et al. concluded that lower pre-transplant CMV antibody titer was significantly associated with CMV infection after kidney and liver transplantation, respectively. Additionally, the quantitative measurement of CMV-specific humoral immunity may play a role in improving CMV prevention strategies in CMV-seropositive organ recipients [29,30].

This study has several strengths. Firstly, we focused on the significance of histologically proven CMV colitis patients no matter whether serum CMV DNA was detectable or not at the time the disease developed. Secondly, our study found that patients with CMV colitis may have negative CMV PCR DNA amplification assays in serum. Most importantly, this is the first study to provide detailed information in terms of clinical characteristics, pre-transplant and post-transplant serology tests from donors and recipients, colonoscopy, and PET/CT images at diagnosis in CMV colitis patients receiving LDLT.

Our study has some limitations. First, due to the rarity and potential problem of underdiagnosis, only two biopsy-proven CMV colitis patients with a history of LDLT were discussed. Second, the fluctuation of the CMV immunoglobulin IgM and IgG levels was not recorded due to some data being unavailable in this retrospective study. Third, all cases were confirmed via positive IHC staining in histology; thus, quantitative PCR in formalin-fixed, paraffin-embedded colon biopsy tissues was not performed. According to previous research, qPCR has the same sensitivity, specificity, and positive/negative predictive value as IHC staining [31,32]. The use of PCR on formalin-fixed, paraffin-embedded tissue has been suggested when IHC staining is negative and there remains a strong clinical suspicion of CMV infection [33]. Additionally, while the endoscopic biopsy test is often used to confirm CMV colitis, the procedure is invasive, and the histological data can be difficult to interpret. Some other non-invasive testing methods such as the detection of CMV DNA in stool or urine samples as well might be clinically helpful. Therefore, a sensitive, specific, and non-invasive test is of more significant interest to the field of liver transplantation. In the future, multicenter prospective studies with a larger sample size of CMV colitis patients in LDLT would be beneficial for clinicians.

In conclusion, patients with immunocompromised status, particularly ones who have undergone organ transplantation, may have positive serum anti-CMV IgM/IgG antibodies both before and after transplantation. Serum anti-CMV IgM, IgG, and CMV DNAemia may not be accurate diagnostic methods for CMV colitis. This study emphasized that endoscopic biopsy with IHC staining may be more powerful for giving an accurate diagnosis of CMV colitis in the setting of living donor liver transplantation.

## Figures and Tables

**Table 1:** Patient profiles of all the biopsy-proven CMV colitis cases (n = 55).

Category	All Patients (n = 55)
Age, mean ± SD (range) (years)	68.0 ± 15.68 (17–97)
Gender	
Male, n (%)	28 (50.9)
Female, n (%)	27 (49.1)
Clinical symptoms	
Bloody stool, n (%)	35 (63.7)
Diarrhea, n (%)	13 (23.6)
Abdominal pain, n (%)	10 (18.2)
Fever, n (%)	8 (14.5)
Lesion site	
Cecum, n (%)	8 (14.5)
Ascending colon, n (%)	8 (14.5)
Transverse colon, n (%)	5 (9.1)
Descending colon, n (%)	11 (20)
Sigmoid colon, n (%)	12(21.8)
Rectum, n (%)	24 (43.6)
Serum laboratory test	
N/L ration, mean ± SD	10.80 ± 20.70
Anti-CMV IgM	
Positive, n (%)	3 (5.5)
Negative, n (%)	22 (40)
Not available, n (%)	30 (54.5)
Anti-CMV IgG	
Positive, n (%)	21 (38.2)
Negative, n (%)	0 (0)
Not available	34 (61.8)
CMV PCR DNA amplification assays	
Positive, n (%)	15 (27.3)
Negative, n (%)	15 (27.3)
Not available, n (%)	25 (45.4)
Pathology	
Inclusion bodies, n (%)	32 (58.2)
CMV IHC staining, n (%)	55 (100)
Anti-viral therapy	
Ganciclovir, n (%)	17 (30.9)
Valganciclovir, n (%)	12 (21.8)
Ganciclovir plus Valganciclovir, n (%)	10 (18.2)
No treatment, n (%)	16 (29.1)
Complications	
Perforation, n (%)	2 (3.6)

**Table 2:** Comparison of biopsy-proven CMV colitis patients with and without CMV DNAemia.

Category	CMV DNAemia (n = 15)	CMV Non-DNAemia (n = 15)	*p* Value
Age, mean ± SD (range) (years)	62.6 ± 19.9	64.5 ± 16.3	0.77
Gender			0.20
Male, n (%)	6 (40)	9 (60)	
Female, n (%)	9 (60)	6 (40)	
Underlying disease			
Ulcerative colitis, n (%)	5 (33)	5 (33)	1.00
Sepsis, n (%)	4 (26.7)	3 (20)	1.00
ESRD, n (%)	1 (6.7)	1 (6.7)	1.00
Chemotherapy for cancer, n (%)	4 (26.7)	1 (6.7)	0.33
AIDS, n (%)	0 (0)	4 (20)	0.10
DM, n (%)	0 (0)	2 (13.3)	0.48
LDLT, n (%)	1 (6.7)	1 (6.7)	1.00
SLE, n (%)	0 (0)	0 (0)	1.00
Clinical symptoms			
Bloody stool, n (%)	7 (46.7)	6 (40)	1.00
Diarrhea, n (%)	4 (26.7)	4 (26.7)	1.00
Abdominal pain, n (%)	6 (40)	3 (20)	0.43
Fever, n (%)	1 (6.7)	2 (13.3)	1.00
Lesion site			
Cecum, n (%)	3 (20)	1 (6.7)	0.60
Ascending colon, n (%)	1 (6.7)	1 (6.7)	1.00
Transverse colon, n (%)	1 (6.7)	2 (13.3)	1.00
Descending colon, n (%)	3 (20)	4 (26.7)	1.00
Sigmoid colon, n (%)	4 (26.7)	4 (26.7)	1.00
Rectum, n (%)	6 (40)	7 (46.7)	1.00
Serum laboratory test			
N/L ratio, mean ± SD	13.2 ± 24.5	3.5 ± 2.9	0.14
Anti-CMV IgM			
Positive, n (%)	2 (13.3)	1 (6.7)	1.00
Negative, n (%)	9 (60)	9 (60)	1.00
Not available, n (%)	4 (26.7)	5 (33.3)	1.00
Anti-CMV IgG			
Positive, n (%)	8 (53.3)	9 (60)	1.00
Negative, n (%)	0 (0)	0 (0)	1.00
Not available, n (%)	7 (46.7)	6 (40)	1.00
Pathology			
Inclusion bodies, n (%)	11 (73.3)	9 (60)	0.7
CMV IHC staining, n (%)	15 (100)	15 (100)	1.00
Anti-viral therapy			
Ganciclovir, n (%)	5 (33.3)	5 (33.3)	1.00
Valganciclovir, n (%)	3 (20)	4 (26.7)	1.00
Ganciclovir plus Valganciclovir, n (%)	5 (33.3)	3 (20)	0.68
No treatment, n (%)	2 (13.3)	3 (20)	1.00

**Table 3:** Comparison of biopsy-proven CMV colitis patients with and without LDLT.

Category	Subgroups of Non-LDLT							All Non-LDLT (n = 53)	LDLT (n = 2)	*p* Value
	Sepsis (n = 13)	UC (n = 12)	ESRD (n = 11)	DM (n = 9)	Chemotherapy * (n = 8)	AIDS (n = 3)	SLE (n = 1)			
Age, mean ± SD (range) (years)	80.5 ± 10.7	63.9 ± 17.5	68.2 ± 7.5	70.6 ± 6.9	61.3 ± 18.4	40.3 ± 14.1	69	68.2 ± 15.9	63.0 ± 32.0	0.38
Gender										
Male, n (%)/Female, n (%)	6 (46.2)/7 (53.8)	5 (41.7)/7 (58.3)	5 (45.5)/6 (54.5)	3 (33.3)/6 (66.7)	7 (87.5)/1 (12.5)	3 (100.0)/0 (0)	0 (0)/1 (100)	27 (51.0)/26 (49.0)	1 (50.0)/1 (50.0)	1.00
Cancer history, n (%)	1 (7.7)	0 (0)	0 (0)	0 (0)	8 (100)	0 (0)	0 (0)	9 (17.0)	1 (50.0)	0.33
Clinical symptoms										
Bloody stool, n (%)	6 (46.2)	8 (66.7)	9 (81.8)	7 (77.8)	4 (50)	0 (0)	1 (100)	35 (66.0)	0 (0)	0.13
Diarrhea, n (%)	5 (38.5)	1 (8.3)	2 (18.2)	1 (11.1)	1 (12.5)	2 (66.7)	0 (0)	12 (22.6)	1 (50)	1.00
Abdominal pain, n (%)	1 (7.7)	2 (16.7)	0 (0)	2 (22.2)	2 (25)	1 (33.3)	0 (0)	8 (15.1)	2 (100)	1.00
Fever, n (%)	2 (15.4)	2 (16.7)	1 (9.1)	1 (11.1)	2 (25)	0 (0)	0 (0)	8 (15.1)	0 (0)	1.00
Lesion site										
Cecum, n (%)	3 (23.1)	0 (0)	1 (9.1)	0 (0)	4 (50)	0 (0)	0 (0)	8 (15.1)	0 (0)	1.00
Ascending colon, n (%)	2 (15.4)	1 (8.3)	0 (0)	0 (0)	3 (37.5)	1 (33.3)	0 (0)	7 (13.2)	1 (50)	1.00
Transverse colon, n (%)	1 (7.7)	0 (0)	1 (9.1)	1 (11.1)	0 (0)	0 (0)	0 (0)	3 (5.7)	2 (100)	0.11
Descending colon, n (%)	2 (15.4)	2 (16.7)	2 (18.2)	1 (11.1)	2 (25)	0 (0)	1 (100)	10 (18.9)	1 (50)	1.00
Sigmoid colon, n (%)	2 (15.4)	5 (41.7)	1 (9.1)	2 (22.2)	1 (12.5)	0 (0)	0 (0)	11 (20.8)	1 (50)	1.00
Rectum, n (%)	4 (30.8)	4 (33.3)	6 (54.5)	5 (55.6)	3 (37.5)	2 (66.7)	0 (0)	24 (45.3)	0 (0)	1.00
Serum laboratory test										
N/L ratio, mean ± SD	14.9 ± 23.9	11.1 ± 25.9	6.8 ± 4.8	15.5 ± 30.7	6.9 ± 4.9	3.1 ± 2.4	3.6	11.1 ± 21.0	1.96 ± 1.5	0.13
Anti-CMV IgM										
Positive, n (%)	0 (0)	2 (16.7)	1 (9.1)	1 (11.1)	0 (0)	0 (0)	0 (0)	3 (5.7)	0 (0)	1.00
Negative, n (%)	5 (38.5)	7 (58.3)	1 (9.1)	2 (22.2)	3 (37.5)	1 (33.3)	1 (100)	20 (37.7)	2 (100)	0.16
Not available, n (%)	8 (61.5)	3 (25)	9 (81.8)	6 (66.7)	5 (62.5)	2 (66.7)	0 (0)	30 (56.6)	0 (0)	0.20
Anti-CMV IgG										
Positive, n (%)	5 (38.5)	6 (50)	1 (9.1)	2 (22.2)	3 (37.5)	1 (33.3)	1 (100)	19 (35.8)	2 (100)	0.14
Negative, n (%)	0 (0)	0 (0)	0 (0)	0 (0)	0 (0)	0 (0)	0 (0)	0 (0)	0 (0)	1.00
Not available	8 (61.5)	6 (50)	10 (90.9)	7 (77.8)	5 (62.5)	2 (66.7)	0 (0)	34 (64.2)	0 (0)	0.14
CMV PCR DNA amplification assays										
Positive, n (%)	4 (30.8)	5 (41.7)	1 (9.1)	0 (0)	4 (50)	0 (0)	0 (0)	14 (26.4)	1 (50)	0.47
Negative, n (%)	2 (15.4)	5 (41.7)	0 (0)	2 (22.2)	3 (37.5)	3 (100)	1 (100)	14 (26.4)	1 (50)	0.47
Not available, n (%)	7 (53.8)	2 (16.7)	10 (90.9)	7 (77.8)	1 (12.5)	0 (0)	0 (0)	25 (47.2)	0 (0)	0.49
Pathology										
Inclusion bodies, n (%)	8 (61.5)	5 (41.7)	5 (45.5)	6 (66.7)	4 (50)	1 (33.3)	1 (100)	30 (56.6)	2 (100)	0.50
CMV IHC staining, n (%)	13 (100)	12 (100)	11 (100)	9 (100)	8 (100)	3 (100)	1 (100)	53 (100)	2 (100)	1.00
Anti-viral therapy										
Ganciclovir, n (%)	4 (30.8)	4 (33.3)	3 (27.3)	2 (22.2)	1 (12.5)	2 (66.7)	1 (100)	17 (32.1)	0 (0)	1.00
Valganciclovir, n (%)	4 (30.8)	5 (41.7)	2 (18.2)	1 (11.1)	4 (50)	0 (0)	0 (0)	12 (22.6)	0 (0)	1.00
Ganciclovir plus Valganciclovir, n (%)	2 (15.4)	2 (16.7)	2 (18.2)	1 (11.1)	1 (12.5)	0 (0)	0 (0)	8 (15.1)	2 (100)	0.04
No treatment, n (%)	3 (23.1)	1 (8.3)	4 (36.4)	5 (55.6)	2 (25)	1 (33.3)	0 (0)	16 (30.2)	0 (0)	1.00
Complications										
Perforation, n (%)	0 (0)	0 (0)	0 (0)	0 (0)	2 (25)	0 (0)	0 (0)	2 (3.8)	0 (0)	1.00

*: chemotherapy for colon cancer (n = 2, 3.6%), cancer of non-gastrointestinal tract (n = 6, 10.9%).

**Table 4:** Profiles of the 2 recipients with biopsy-proven CMV colitis after living donor liver transplantation.

Category	Recipient No. 1	Recipient No. 2
Age (years)	67	59
Gender	Male	Female
Etiology of underlying liver disease	Non-B, non-C liver cirrhosis	Angiosarcoma, ruptured
Clinical symptoms	Abdominal pain, diarrhea	Abdominal pain
Lesion site	Transverse colon Descending colon	Ascending colon Transverse colon Sigmoid colon
Colonoscopy findings	Segmental ulcer with intra-luminal narrowing	Patchy ulcers
Serum laboratory test		
*Recipient*		
Anti-CMV IgM		
Pre-transplant, Index	Negative (0.08)	Negative (0.19)
Post-transplant (at time of disease), Index	Negative (0.06)	Negative (0.34)
Anti-CMV IgG		
Pre-transplant, AU/mL	Positive (2115.3)	Positive (73.6)
Post-transplant (at time of disease), AU/mL	Positive (1199.3)	Positive (2280.9)
CMV-DNA PCR (at time of disease) IU/mL	Not detectable	Positive (123)
WBC (at time of disease) 1000/uL	16.2	6.2
CRP (at time of disease) mg/L	89.72	9.7
N/L ratio	2.99	0.93
*Donor*		
Anti-CMV IgM (Index)	Negative (0.08)	Negative (0.06)
Anti-CMV IgG (AU/mL)	Positive (615.3)	Negative (1.8)
CMV-DNA PCR (IU/mL)	Not available	Not available
Pathology		
Inclusion bodies	Present	Present
CMV IHC staining	Positive	Positive
Anti-viral medication		
Ganciclovir	14 days	12 days
Valganciclovir	90 days	160 days
Anti-viral medication duration (days)	104 days	172 days
Time to disease	10 years after LDLT	2 months after LDLT
Immunosuppressants use at time of CMV colitis	Mycophenolate mofetil 250mg Q12H PO, Tacrolimus 1mg QD PO	Mycophenolate mofetil 500 mg Q12H PO Prednisolone 5mg TID PO Tacrolimus 2mg QD PO
Post-LDLT outcomes		
Allograft dysfunction	No	No
Biliary tract stricture	No	Present
Acute cellular rejection	Yes	No

## Data Availability

Due to the participant consent obtained as part of the recruitment process, it is not possible to make these data publicly available. The data presented in this study are available on request from the corresponding author.
